# Pathology and Treatment of Simple Fracture of the Patella

**Published:** 1904-09

**Authors:** 


### PATHOLOGY AND TREATMENT OF SIMPLE FRACTURE OF THE PATELLA.

E. Eliot (*Medical News*, June 11, 1904) says that owing to the not infrequent uncontrollable rotation of the lower fragment through an arc of 90 degrees on its horizontal axis accurate apposition of the fracture surfaces can not always be secured, and under these circumstances the ultimate result of conservative treatment could scarcely prove satisfactory. Suitable apposition may also be prevented by the interposition of torn shreds of capsule, by extravasated blood-clot, and possibly also by the interposition of untorn periosteum, conditions which can be recognized and remedied by exploration. Suture of the fragments permits of simultaneous suture of the torn capsule, a procedure of the greatest importance for restoration of joint security. The results of suture are almost invariably excellent, and the risk of operation is comparatively slight. The writer declares that in no way does he wish to belittle the risk of operation. The exercise of the most rigid aseptic precautions in all that pertains to the operation can not be too greatly emphasized. If in any case there are reasons why the highest degree of surgical asepsis can not be practiced, it is far better to advise some non-operative procedure than to subject the patient to the possibility of a septic arthritis.

